# Prevalence of loneliness and its associated factors in middle-aged and older adults with breathlessness: a nationally representative cohort study

**DOI:** 10.1136/bmjresp-2026-004357

**Published:** 2026-07-29

**Authors:** Ruth A Hackett, Sam Norton, Joseph Chilcot, Laura Cottrell, Matthew Maddocks, Irene J Higginson, Martha Canfield, Keir E J Philip, Lisa Jane Brighton

**Affiliations:** 1 Department of Psychology, Institute of Psychiatry Psychology & Neuroscience, King’s College London, London, UK; 2 Cicely Saunders Institute of Palliative Care, Policy & Rehabilitation, King’s College London, London, UK; 3 Department of Psychology, Glasgow Caledonian University, Glasgow, UK; 4 National Heart and Lung Institute, Imperial College London, London, UK

**Keywords:** Clinical Epidemiology

## Abstract

**Background:**

Breathlessness has functional, psychological and social impacts. Loneliness is a health priority under-researched in relation to breathlessness, despite breathlessness impacting social interactions.

**Methods:**

We estimated the prevalence of loneliness and associated characteristics in adults (≥50 years) with breathlessness in the English Longitudinal Study of Ageing (ELSA, 2010–11). Breathlessness was assessed using the adapted modified Medical Research Council Dyspnoea Scale from grade 0 (none) to 3 (stops on level due to breathlessness). Grade 4 was not assessed in ELSA. Loneliness was measured using the 3-Item University of California, Los Angeles Loneliness Scale (scores ≥6 = lonely). Logistic regression was adjusted for age, sex and ethnicity.

**Results:**

Of the 8195 participants, 28.4% reported breathlessness and 19.3% were lonely. Greater breathlessness was associated with older age, female sex and lower wealth (p<0.001). Those with greater breathlessness were more likely to be lonely (16.3% for no breathlessness vs 34.9% for grade 3), live alone (18.2% vs 36.6%) and be unmarried (26.7% vs 45.7%) (all p<0.001). Greater breathlessness was associated with depressive symptoms, limiting longstanding illness, mobility issues (all p<0.001) and social isolation (p=0.012). Of those with worse breathlessness (graded 2–3, n=1149), 32.8% were lonely. Characteristics associated with loneliness in those with grade 2–3 breathlessness were female sex (OR 1.32, 95% CI 1.02 to 1.71), lower wealth (OR 2.75, 95% CI 1.63 to 4.64), no qualifications versus having a degree (OR 1.65, 95% CI 1.03 to 2.66), being unmarried (OR 3.45, 95% CI 2.64 to 4.52), living alone (OR 3.71, 95% CI 2.80 to 4.91), depressive symptoms (OR 1.58, 95% CI 1.47 to 1.69), limiting longstanding illness (OR 1.76, 95% CI 1.30 to 2.38), smoking (OR 1.42, 95% CI 1.04 to 1.97), difficulties with activities of daily living (OR 1.34, 95% CI 1.21 to 1.47) or mobility (OR 1.15, 95% CI 1.10 to 1.21). Age, ethnicity or social isolation was not significantly associated with loneliness in adults with breathlessness.

**Conclusions:**

Adults with breathlessness are more likely to be lonely, especially those with lower socio-economic status, who are unmarried or live alone or who have poorer physical function. Findings highlight priority groups for loneliness interventions among adults with breathlessness.

**WHAT IS ALREADY KNOWN ON THIS TOPIC**

- Loneliness is a major public health concern in middle-aged and older adults, associated with poor physical health and premature mortality. While other symptoms such as pain, fatigue and depression are known contributors to loneliness, breathlessness has been largely absent from the loneliness literature, with the limited existing evidence restricted to small studies or populations with self-reported chronic obstructive pulmonary disease.

**WHAT THIS STUDY ADDS**

- Using a large, nationally representative sample, this study demonstrates that loneliness increases progressively with breathlessness severity, from 16.3% in those with no breathlessness to 34.9% in those with modified Medical Research Council (mMRC) grade 3 breathlessness. Among adults with mMRC grade 2–3 breathlessness, the characteristics associated with loneliness included being unmarried or living alone, lower socio-economic status, depressive symptoms, limiting longstanding illness and mobility impairment.

**HOW THIS STUDY MIGHT AFFECT RESEARCH, PRACTICE OR POLICY**

- Adults living with breathlessness should be considered a high-risk group for loneliness screening and intervention, particularly those who are socio-economically disadvantaged, living alone or experiencing comorbid depressive symptoms or functional impairment. Future research should use longitudinal designs to examine the mechanisms linking breathlessness to loneliness.

## Introduction

 Loneliness is a public health concern, increasingly described as an ‘epidemic’.^1^ Loneliness is the perceived lack of companionship, arising from a mismatch between actual and desired social connection.^2^ The number of people aged >50 experiencing loneliness in England is predicted to reach 2 million by 2026, an increase of 49% since 2017.^3^ Loneliness is associated with depression, poorer physical health and premature mortality,^4–7^ alongside significant economic consequences from lost productivity and increased healthcare utilisation.^8^

Breathlessness is a common and distressing symptom experienced by up to a third of adults in the general population^9 10^ and up to 48.6% of clinical patient populations.^10^ It accompanies a wide range of chronic conditions including lung disease, heart disease, cancer and neurological conditions.^11 12^ Breathlessness often worsens over time, becoming chronic and persistent despite optimal disease management.^13^ People living with breathlessness typically face a shrinking social world, with symptoms that disrupt verbal communication, restrict mobility and limit access to services, all compounded by stigma arising from perceptions of self-blame, contributing to the pervasive invisibility of this population.^14–17^

Despite these features, breathlessness remains largely absent from the loneliness literature, and loneliness is not featured in recent reviews of breathlessness correlates.^10 18^ Other multidimensional symptoms, including pain, depression and fatigue, are known to contribute to loneliness,^19^ yet equivalent evidence for breathlessness is sparse, with existing studies generally small in scale or largely limited to self-reported chronic obstructive pulmonary disease (COPD) in population samples.^20^ Quantifying loneliness prevalence across breathlessness grades identifies burden, and profiling the characteristics of those experiencing both loneliness and breathlessness could inform screening and targeted interventions, aligning with WHO priorities on loneliness in ageing^21^ and the UK Government Loneliness Strategy’s focus on those with physical ill-health.^8^

This study therefore aimed to determine the prevalence of, and factors associated with, loneliness in middle aged and older people with breathlessness. Specific objectives were to (i) estimate the prevalence of loneliness in middle aged and older people across the modified Medical Research Council (mMRC) grades of breathlessness (0 = no breathlessness to grade 3 = stops walking on level due to breathlessness) and (ii) explore demographic and clinical characteristics associated with loneliness in people with more severe breathlessness (mMRC grades 2–3) where we hypothesise activity limitations may impair social functioning and contribute to loneliness.

## Materials and methods

### Study population

We used data from the English Longitudinal Study of Ageing (ELSA), a longitudinal cohort study of community dwelling adults aged ≥50 years (known as ELSA core participants) and their partners in England.^22^ ELSA data collection began in 2002 (wave 1), with follow-up waves biennially. At every wave, data are collected via computer-assisted personal interview and self-reported questionnaires. Anthropometric and clinical data are gathered at alternate waves. ELSA received ethical approval from the London Multicentre Research and Ethics Committee (MREC/01/02/91) in accordance with the Declaration of the World Medical Association. Participants provided fully informed consent.

**Figure 1:**
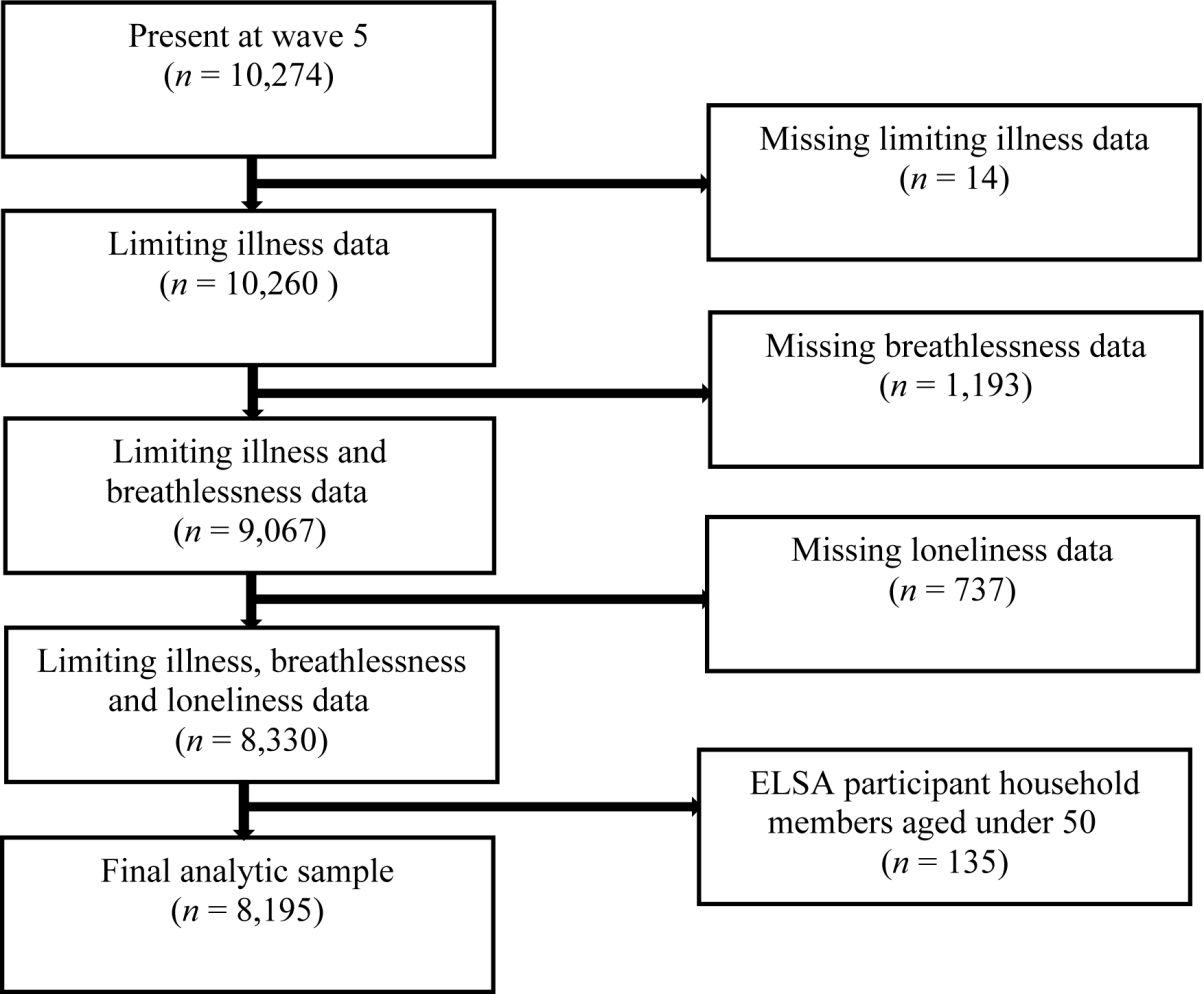
Flowchart of participants included and excluded from the study at wave 5 of the English Longitudinal Study of Ageing.

In this study, we used cross-sectional data from wave 5 (2010–2011), the last wave when information on breathlessness was collected. To address our research objectives, the sample was restricted to those aged ≥50 with complete information on loneliness, breathlessness and reports of limiting longstanding illness. A flowchart of those included and excluded from the study can be found in figure 1. A total of 10 274 adults participated in wave 5 of ELSA. From this sample, 8330 participants provided complete data on loneliness, breathlessness and limiting longstanding illness. Of these 8330 participants, 8195 were aged ≥50. These 8195 adults constitute the final analytic sample for this study. All analyses were conducted as complete-case analyses.

Compared with those excluded from the analysis (n=2079), the included sample (n=8195) were significantly younger on average (66.11 vs 68.93 years, p<0.001) and were less likely to be from an ethnic minority background (2.6% vs 6.8%, p<0.001). The included group was more likely to be married (69.7% vs 58.7%, p<0.001) and less likely to be lonely (19.3% vs 37.0%, p<0.001) and to live alone (21.8% vs 31.3%, p<0.001) than the excluded group. However, the excluded group was less likely to be socially isolated than the included group (no social isolation, 71.0% vs 14.1%, p<0.001). The included sample was significantly wealthier (highest wealth quintile, 22.9% vs 12.6%, p<0.001) and had lower depressive symptom scores (1.36 vs 2.42, p<0.001) on average than the excluded group. The included group was less likely to have a limiting longstanding illness (30.2% vs 55.0%, p<0.001) and report high levels of breathlessness (grade 3 breathlessness, 9.5% vs 16.2%, p<0.001). The included group had fewer mobility impairments on average (1.61 vs 3.44, p<0.001) and was less likely to be current smokers (12.1% vs 18.2%, p<0.001). The included and excluded groups did not significantly differ by sex (p=0.108).

### Patient and public involvement

Findings were shared with patient and public advisory group members for feedback on interpretation.

### Measures

#### Loneliness

Our main loneliness measure was the three-item revised University of California, Los Angeles (UCLA) loneliness scale.^23^ At wave 5, participants rated items such as “How often do you feel you lack companionship*?”* with response options of 1 = *hardly ever/never*, 2 = *some of the time* and 3 = *often*. Scores on the scale ranged from 3 to 9, with higher values indicating greater loneliness. In line with previous work in the ELSA cohort,^24 25^ scores ≥6 were used to define high levels of loneliness. A one-item measure of loneliness was also collected at wave 5 asking respondents, *“*How often do you feel lonely*?”* with response options of 1 = *hardly ever/never*, 2 = *some of the time* and 3 = *often*. This single item was used to derive a binary variable where 0 = *hardly ever or never/some of the time* and 1 = *often*. The three-item UCLA scale was used as the primary loneliness measure throughout, as it captures multiple facets of perceived social deficit. The single-item measure is reported descriptively across breathlessness grades only to allow comparison with literature using single-item measures.

#### Breathlessness

Breathlessness was assessed using the mMRC Dyspnoea Scale, a widely used scale in the field^10^ as recommended by the National Institute for Health and Care Excellence.^26^ Participants report their experiences of breathlessness on exertion (no/yes) using a 4-point scale: grade 0 = *no breathlessness on exertion*, grade 1 = *breathless when walking up a slight hill or when hurrying on the level*, grade 2 = *breathless when walking with people of own age at own pace on the level* and grade 3 = *respondent has to stop due to breathlessness while walking at own pace on the level*. The full mMRC scale usually contains a grade 4 = *too breathless to leave the house or breathless when dressing/undressing*. However, this item was not included in the ELSA version of this questionnaire.

#### Limiting longstanding illness

Self-reported limiting longstanding illness was assessed with two questions: (1) “Do you have any long-standing illness, disability, or infirmity? By long-standing I mean anything that has troubled you over a period of time or that is likely to affect you over a period of time*”*. If the participant responded yes, they were asked (2) *“*Does this illness or disability limit your activities in any way*?”*. If the participant responded yes to both items, they were classified as having a limiting longstanding illness (no/yes) as in previous work.^27^

#### Demographic measures

All demographic measures were assessed at wave 5. Participants self-reported their age (years), sex (male/female) and ethnicity (white/mixed ethnicity/black/Asian/other). Two measures of socio-economic position were included: household non-pension wealth and self-reported education. Household non-pension wealth is the most relevant indicator of socio-economic position in this cohort^22^ and is divided into quintiles across the entire wave five sample (1 = lowest wealth; 5 = highest wealth). Education was derived as a three-category variable: 0 = no educational qualifications, 1 = A-levels/0-levels or equivalent and 2 = university degree.

#### Measures of social connectedness

Participants self-reported their marital status (married/single/separated/divorced/widowed) and whether they lived alone (no/yes). Social isolation was derived as an index based on the extent of contact within a participant’s social network and their involvement with social organisations.^4 28^ They were asked about frequency of contact with their children, other family and friends with response options of *less than once a year/never, once or twice a year, every few months, once or twice a month, once or twice a week* and *three or more times a week*. They received a point if they had less than monthly face-to-face or telephone contact with each of the three categories of social tie. Participants received another point if they did not participate in any social organisation (eg, social or sports clubs, churches or residents’ groups). Total scores ranged from 0 to 4, with higher scores indicating greater isolation. Few participants received a score of 4, so categories 3 and 4 were combined.

#### Measures of health and well-being

Depressive symptoms were measured using the 8-item Center for Epidemiologic Studies-Depression (CES-D) scale,^29^ in which higher scores indicate severe symptoms. Items included statements, such as “I felt depressed*”* and “My sleep was restless*”* (scores ranging from 0 to 8). As a sensitivity check, the CES-D item on loneliness was excluded to avoid direct overlap with the UCLA loneliness scale in line with other ELSA research.^4 30^ A dichotomous response to each item resulted in a total score ranging from 0 to 7. Smoking behaviour was categorised as a two-level variable (non-smokers or current smokers). Participants self-reported whether they had a history or a current doctor diagnosis of a range of physical health conditions. Participants self-reported angina or myocardial infarction, and this information was used to generate a measure of coronary heart disease (no/yes).^31^ They also self-reported diagnoses of stroke (no/yes), diabetes (no/yes), lung disease such as chronic bronchitis or emphysema (no/yes), asthma (no/yes), arthritis (no/yes) and cancer (no/yes). Self-reported diagnoses are presented for descriptive purposes only and not included in regression models. Consistent with the symptom-based focus of this study, limiting longstanding illness was used as a single measure of chronic illness in regression models, rather than individual disease indicators.

#### Measures of mobility and impairment

Mobility impairment was assessed by asking respondents if they had any difficulty performing 10 everyday activities (eg, walking 100 yards and getting up from a chair after sitting for long periods) because of a health problem (no/yes). Scores on this measure ranged from 0 to 10. Activities of daily living (ADL) impairment was assessed with six items asking participants whether a health or memory problem caused them difficulty with the following activities: dressing, walking across a room, bathing or showering, eating, getting in or out of bed and using the toilet, with response options of no/yes.^32^ Scores on the ADL measure ranged from 0 to 6. Impairment with instrumental ADL (IADLs), which relate to having an independent lifestyle, was also assessed. Nine IADL impairments were assessed with response options of no/yes: difficulty using a map to figure out how to get around in a strange place, recognising physical danger, preparing a hot meal, shopping for groceries, making telephone calls, communication, taking medications and doing work around the house or garden and financial management.^33^ Scores on the IADL measure ranged from 0 to 9.

#### Statistical analyses

Participant characteristics across the entire sample (n=8195) are presented as means (SD) for continuous variables and number (percentage) for categorical variables. Demographic and clinical characteristics across the four grades of breathlessness were compared using univariate analysis of variance for continuous variables and χ2 tests for categorical variables. These analyses were repeated, applying cross-sectional sample weights of ELSA wave 5 (*w5xwgt*) to adjust for non-response and sample attrition from earlier ELSA waves. This weight is used to ensure representativeness of the English household population aged ≥50 years.^34^ Only ELSA core participants (ELSA longitudinal participants excluding life partners) have sample weights, which reduced our sample size for these analyses (n=7307).

To maximise the sample size, sample weights were not included for the main analyses, where unweighted logistic regression analyses were conducted to assess the demographic and clinical characteristics associated with loneliness (0 = scores <6; 1 = scores ≥6) in those with grade 2 and 3 breathlessness (n*=*1149), as ≥2 is the most common criterion for clinically significant breathlessness.^10^ Each candidate characteristic was modelled in a separate logistic regression with loneliness as the outcome, adjusting only for age, sex and ethnicity, selected a priori as established demographic confounders of both loneliness and breathlessness.^9 35 36^ Results are presented as OR and 95% CIs. The association with loneliness (0=scores <6; 1=scores ≥6) in participants without breathlessness (n=5870) was also investigated and compared with those with grade 2 and 3 breathlessness (n*=*1149) using unweighted adjusted logistic regression models.

As loneliness is a common outcome (prevalence >10%) in preliminary analyses, binomial regression was used to obtain risk ratios, as ORs can overestimate associations under these conditions. The use of binomial regression did not change the pattern of results, so these analyses are not presented here. In preliminary analyses, additive interactions were used between breathlessness and age (<60 or ≥60 years), sex and ethnicity using relative excess risk due to interaction (*reri* in Stata). The interactions for age (p=0.094), sex (p*=*0.337) and ethnicity (p=0.337) were not statistically significant and therefore were not included in our main analyses. Analyses were conducted using IBM SPSS Statistics V.24 (IBM Corp., Armonk, NY, USA) and Stata V.18 (StataCorp LLC, College Station, TX, USA).

## Results

### Demographic, health and mobility measures associated with breathlessness

The characteristics of the sample by breathlessness grade are presented in table 1. Of the 8195 participants, 2142 (28.4%) reported some breathlessness (grades 1–3). Participants with greater breathlessness were significantly older, more likely to be female and were less wealthy and less educated on average than those without breathlessness (all p<0.001). No significant difference in ethnicity was observed across the breathlessness groups (p=0.394).

**Table 1:** Characteristics of sample by breathlessness grade at wave 5 (2010/11) of the English Longitudinal Study of Ageing (unweighted)

	Total sample (n=8195)	No breathlessness (n=5870)	Grade 1 breathlessness (n=1176)	Grade 2 breathlessness (n=370)	Grade 3 breathlessness (n=779)	P value
Age (years)	66.11 (8.75)	64.97 (8.40)	68.52 (8.64)	67.99 (8.85)	70.15 (9.33)	**<0.001**
Sex (%, female)	4518 (55.1%)	3062 (52.2%)	755 (64.2%)	248 (67.0%)	453 (58.2%)	**<0.001**
Ethnicity (%, yes)*	–	–	–	–	–	0.394
White	7978 (97.4%)	5725 (97.6%)	1145 (97.4%)	357 (96.6%)	751 (96.4%)	
Mixed ethnicity	16 (0.2%)	10 (0.2%)	3 (0.3%)	2 (0.5%)	1 (0.1%)	
Black	57 (0.7%)	38 (0.6%)	10 (0.8%)	2 (0.5%)	7 (0.9%)	
Asian	104 (1.3%)	65 (1.1%)	16 (1.3%)	7 (1.9%)	16 (2.1%)	
Other	36 (0.4%)	28 (0.5%)	2 (0.2%)	2 (0.5%)	4 (0.5%)	
Wealth quintile (£)†	–	–	–	–	–	**<0.001**
1	1112 (15.2%)	563 (10.9%)	218 (20.1%)	74 (22.0%)	257 (35.4%)	
2	1443 (19.8%)	915 (17.7%)	256 (23.6%)	85 (25.2%)	187 (25.8%)	
3	1478 (20.2%)	1036 (20.1%)	234 (21.6%)	84 (24.9%)	124 (17.1%)	
4	1598 (21.9%)	1241 (24.1%)	204 (18.8%)	58 (17.2%)	95 (13.1%)	
5	1672 (22.9%)	1402 (27.2%)	172 (15.9%)	36 (10.7%)	62 (8.6%)	
Education (%, yes)‡	–	–	–	–	–	**< 0.001**
University degree	1703 (20.9%)	1459 (25.0%)	140 (11.9%)	42 (11.4%)	62 (8.0%)	
A-levels/O-levels	4590 (56.2%)	3331 (57.0%)	669 (57.1%)	213 (57.6%)	377 (48.5%)	
No qualifications	1870 (22.8%)	1054 (18.0%)	363 (31.0%)	115 (31.0%)	338 (43.5%)	
Marital status (%, yes)§	–	–	–	–	–	**<0.001**
Married	5711 (69.7%)	4305 (73.3%)	739 (62.9%)	245 (66.2%)	422 (54.2%)	
Never married	462 (5.6%)	345 (5.9%)	61 (5.2%)	14 (3.8%)	42 (5.4%)	
Divorced	930 (11.3%)	610 (10.4%)	151 (12.9%)	44 (11.9%)	125 (16.0%)	
Widowed	1091 (13.3%)	610 (10.4%)	224 (19.0%)	67 (18.1%)	190 (24.4%)	
UCLA loneliness (SD)	4.11 (1.50)	3.96 (1.39)	4.25 (1.52)	4.51 (1.68)	4.84 (1.88)	**<0.001**
UCLA loneliness ≥6 (%)	1581 (19.3%)	956 (16.3%)	248 (21.1%)	105 (28.4%)	272 (34.9%)	**<0.001**
Loneliness one item (% yes)¶	528 (6.4%)	265 (4.5%)	85 (7.2%)	37 (10%)	143 (18.1%)	**<0.001**
Social isolation (no marital status)**	–	–	–	–	–	**0.012**
0	904 (14.1%)	648 (14.3%)	132 (14.0%)	37 (12.5%)	87 (13.6%)	
1	3490 (54.3%)	2525 (55.5%)	497 (52.9%)	159 (53.7%)	309 (48.3%)	
2	1606 (25.0%)	1097 (24.1%)	245 (26.1%)	79 (26.7%)	185 (28.9%)	
3	425 (6.6%)	279 (6.1%)	66 (7.0%)	21 (7.1%)	59 (9.2%)	
Living alone (% yes)	1785 (21.8%)	1068 (18.2%)	336 (28.6%)	96 (25.9%)	285 (36.6%)	**<0.001**
CES-D depression (SD)††	1.36 (1.85)	1.07 (1.61)	1.58 (1.89)	2.24 (2.17)	2.83 (2.38)	**<0.001**
CES-D (without loneliness) (SD)††	1.25 (1.69)	0.99 (1.48)	1.44 (1.71)	2.06 (1.97)	2.57 (2.13)	**<0.001**
Limiting illness (%, yes)	2472 (30.2%)	1168 (19.9%)	452 (38.4%)	244 (65.9%)	608 (78.0%)	**<0.001**
Coronary heart disease (%, yes)	1019 (12.4%)	450 (7.7%)	217 (18.5%)	82 (22.2%)	270 (34.7%)	**<0.001**
Stroke (%, yes)	275 (3.4%)	124 (2.1%)	45 (3.8%)	25 (6.8%)	81 (10.4%)	**<0.001**
Diabetes (%, yes)	824 (10.1%)	456 (7.8%)	142 (12.1%)	62 (16.8%)	164 (21.1%)	**<0.001**
Lung disease (%, yes)	381 (4.6%)	79 (1.3%)	80 (6.8%)	55 (14.9%)	167 (21.4%)	**<0.001**
Asthma (% yes)	943 (11.5%)	425 (7.2%)	217 (18.5%)	73 (19.7%)	228 (29.3%)	**<0.001**
Arthritis (% yes)	3004 (36.7%)	1765 (30.1%)	536 (45.6%)	226 (61.1%)	477 (61.2%)	**<0.001**
Cancer (% yes)	499 (6.1%)	305 (5.2%)	84 (7.1%)	31 (8.4%)	79 (10.1%)	**<0.001**
Smoking status	–	–	–	–	–	**<0.001**
Non-smoker	7206 (87.9%)	5279 (89.9%)	986 (83.8%)	316 (85.4%)	625 (80.2%)	
Current	989 (12.1%)	591 (10.1%)	190 (16.2%)	54 (14.6%)	154 (19.8%)	
ADL (SD)	0.24 (0.71)	0.11 (0.47)	0.26 (0.66)	0.59 (1.07)	0.99 (1.32)	**<0.001**
IADL (SD)	0.27 (0.77)	0.14 (0.52)	0.28 (0.73)	0.66 (1.07)	1.12 (1.36)	**<0.001**
Mobility issues (SD)‡‡	1.61 (2.25)	0.93 (1.63)	2.10 (2.01)	3.82 (2.48)	4.93 (2.68)	**< 0.001**

Data presented as means (SD) and n (%). Percent is valid percent.

Bolded p values are statistically significant p<0.05.

* n=8191 ethnicity.

† n=7303 wealth.

‡ n=8163 education.

§ n=8194 marital status.

¶ n=8188 single-item loneliness.

** n=6425 social isolation.

†† n=8165 CES-D.

‡‡ n=8194 Mobility issues.

ADL, activities of daily living; CES-D, Centre for Epidemiologic Studies Depression Scale; IADL, instrumental activities of daily living; UCLA, University of California, Los Angeles Scale.

For the measures of health and well-being, greater breathlessness was associated with significantly higher depressive symptoms on the full CES-D scale (p<0.001) and when the loneliness item was removed from this scale (p<0.001). Those reporting more breathlessness had a significantly greater likelihood of limiting longstanding illness (19.9% for no breathlessness vs 78.0% for grade 3, p<0.001) than those without breathlessness, including coronary heart disease, diabetes, lung disease, asthma, arthritis and cancer (all p<0.001). Those reporting higher grades of breathlessness were also more likely to be current smokers than those without breathlessness (p<0.001).

In terms of measures of mobility and impairment, those reporting greater breathlessness had significantly more mobility issues (p<0.001), as well as greater difficulties with ADLs and IADLs than those without breathlessness. When these analyses were repeated, applying sample weights to adjust for non-response and attrition (n=7307), the pattern of results was unchanged (online supplemental table 1).

### Association of loneliness and breathlessness

**Figure 2:**
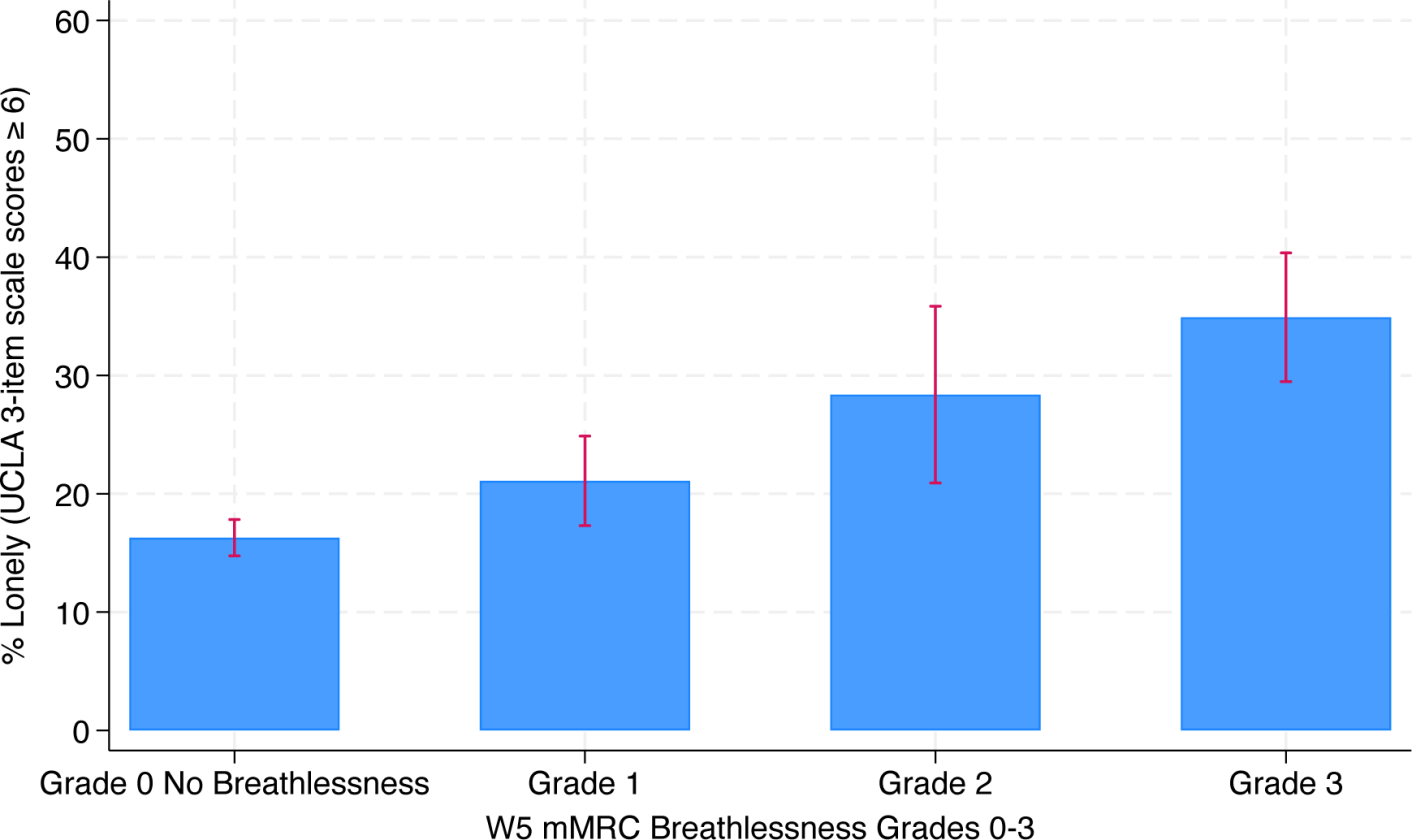
Unadjusted prevalence of loneliness by breathlessness group. Unadjusted prevalence of loneliness (scores ≥6 on the UCLA loneliness scale) by breathlessness group at wave 5 of the English Longitudinal Study of Ageing. Error bars are 95% CIs. mMRC, modified Medical Research Council; UCLA, University of California, Los Angeles.

In the unadjusted analyses (table 1), 19.3% (n=1581) of the sample were lonely (scores ≥6 on the UCLA loneliness scale). Participants with greater breathlessness were more likely to be lonely (no breathlessness = 16.3% vs grade 3 = 34.9%, χ²(3) =178.26, p<0.001). This unadjusted relationship of loneliness prevalence by mMRC grade is graphically represented in figure 2. They also had higher loneliness scores on the continuous UCLA scale (no breathlessness, mean=3.96, SD=1.39; grade 3, mean=4.84, SD=1.88; F(3,8191)=95.49, p<0.001) and on the single item loneliness measure (no breathlessness, 4.5%; grade 3, 18.1%; χ²(3)=221.56, p<0.001). On other measures of social connectedness, those with greater breathlessness were more likely to live alone (no breathlessness, 18.2%; grade 3, 36.6%, χ²(3)=180.13, p<0.001), be unmarried (no breathlessness, 26.7%; grade 3, 45.7%, χ²(9)=217.21, p<0.001) and be more socially isolated (χ²(9)=21.17, p=0.012). When these analyses were repeated, applying sample weights (n=7307), the pattern of results was similar (online supplemental table 1).

### Characteristics associated with loneliness in those with breathlessness

Of those with chronic breathlessness (grade 2 and 3 breathlessness, n=1149), 32.8% were lonely. In the adjusted analyses (table 2), the odds of loneliness were significantly higher (OR 2.47, 95% CI 2.13 to 2.85, p<0.001) in those with grade 2 and 3 breathlessness compared with people without breathlessness (n=5870; reference group for analysis).

**Table 2:** Association with loneliness comparing people without breathlessness (n=5870) with those with grade 2 and 3 breathlessness (n*=*1149) at wave 5 (2010/11) of the English Longitudinal Study of Ageing

	OR (95% CI)	P value
Breathlessness (reference: no breathlessness)	2.47 (2.13 to 2.85)	<0.001

Analysis is adjusted for age, sex and ethnicity.

Logistic regression analyses assessing the characteristics associated with loneliness in participants with breathlessness after adjusting for age, sex and ethnicity can be found in table 3. The demographic characteristics associated with loneliness in the breathlessness group were female sex (OR 1.32, 95% CI 1.02 to 1.71, p=0.033), lower versus higher wealth (OR 2.75, 95% CI 1.63 to 4.64, p<0.001) and having no qualifications versus having a degree (OR 1.65, 95% CI 1.03 to 2.66, p=0.039). Loneliness was not significantly associated with age (p=0.056) after adjusting for sex and ethnicity in the breathlessness group. It was also not associated with ethnicity (p*=*0.163) in the breathlessness group after adjusting for age and sex.

**Table 3:** Characteristics associated with loneliness in those with grade 2 and 3 breathlessness (n*=*1149) at wave 5 (2010/11) of the English Longitudinal Study of Ageing

	OR (95% CI)	P *value*
Age	0.99 (0.97 to 1.00)	0.056
Sex (reference: male)	1.32 (1.02 to 1.71)	**0.033**
Ethnicity binary (reference: white)	1.57 (0.83 to 2.96)	0.163
Wealth (reference: highest wealth quintile 5)		**<0.001**
1	2.75 (1.63 to 4.64)	**<0.001**
2	1.68 (0.98 to 2.88)	0.061
3	1.29 (0.73 to 2.28)	0.374
4	1.09 (0.60 to 2.00)	0.778
Education (reference: university degree)		**0.026**
No qualifications	1.65 (1.03 to 2.66)	**0.039**
A-levels/O-levels	1.21 (0.76 to 1.94)	0.420
Marital status binary (reference: married)	3.45 (2.64 to 4.52)	**<0.001**
Living alone (reference: no)	3.71 (2.80 to 4.91)	**<0.001**
Social isolation without marital status	0.99 (0.83 to 1.17)	0.889
CES-D depression	1.59 (1.49 to 1.70)	**<0.001**
CES-D (loneliness item removed)	1.58 (1.47 to 1.69)	**<0.001**
Limiting illness (reference: no)	1.76 (1.30 to 2.38)	**<0.001**
Smoking (reference: non-smoker)	1.42 (1.04 to 1.97)	**0.032**
ADL (reference: no)	1.34 (1.21 to 1.47)	**<0.001**
IADL (reference: no)	1.29 (1.18 to 1.42)	**<0.001**
Mobility issues	1.15 (1.10 to 1.21)	**<0.001**

Each row reports a separate logistic regression model, with the listed characteristic as the sole predictor of interest, adjusted for age, sex and ethnicity.

Bolded p values are statistically significant p<0.05.

ADL, activities of daily living; CES-D, Center for Epidemiologic Studies Depression Scale; IADL, instrumental activities of daily living.

In terms of the social connectedness variables, loneliness was significantly associated with greater odds of being unmarried (OR 3.45, 95% CI 2.64 to 4.52, p<0.001) and living alone (OR 3.71, 95% CI 2.80 to 4.91, p<0.001). No significant associations between loneliness and social isolation were found in the breathlessness group (p=0.889).

For the health and well-being measures, the characteristic associated with loneliness in the breathlessness group were greater depressive symptoms (OR 1.58, 95% CI 1.47 to 1.69, p<0.001), having a limiting longstanding illness (OR 1.76, 95% CI 1.30 to 2.38, p<0.001) and smoking (OR 1.42, 95% CI 1.04 to 1.97, p=0.032). In terms of the mobility and impairment variables, loneliness was significantly associated with greater odds of mobility issues (OR 1.15, 95% CI 1.10 to 1.21, p<0.001), as well as greater difficulties with ADL (OR 1.34, 95% CI 1.21 to 1.47, p<0.001) and IADL (OR 1.29, 95% CI 1.18 to 1.42, p<0.001) in the breathlessness group.

## Discussion

This study used data from a large nationally representative sample of middle-aged and older adults in England to estimate the prevalence of loneliness in people with breathlessness and identify associated characteristics. Nearly one-third of those with moderate to severe breathlessness (mMRC grades 2–3) were lonely, and greater breathlessness was consistently associated with greater loneliness. These findings make an important contribution to an underexplored area, identifying breathlessness potentially as a clinically relevant correlate of loneliness, alongside other somatic symptoms (eg, pain and fatigue) and depression. Given the cross-sectional design, these findings should be interpreted as associations rather than causal or directional relationships.

In our sample of over 8000 participants, one of the largest studies of its kind, demographic and clinical characteristics were assessed across four grades of breathlessness. Nearly a third of the sample reported some degree of breathlessness,^9 10^ underscoring breathlessness as a common symptom at middle and older age. Breathlessness was more frequent among older adults and women.^9 10 18^ Further, a socio-economic gradient emerged: the heaviest burden of breathlessness was found among those in the lowest wealth quintile and those less likely to have a degree. These patterns reveal that breathlessness disproportionately affects lower socio-economic status groups.^10 37 38^

Participants with breathlessness were more likely to report a limiting longstanding illness. The significant associations between breathlessness and a range of chronic conditions, such as heart disease, stroke, diabetes, asthma, arthritis, lung disease and cancer, should be interpreted with caution given the cross-sectional study design. The relationship between breathlessness and these conditions is likely complex and bidirectional.^39^ While breathlessness is frequently a symptom of underlying disease, shared risk factors such as obesity may drive both breathlessness and conditions such as diabetes and arthritis.^40 41^ Similarly, in agreement with review evidence,^10^ those with breathlessness reported increased depressive symptoms. This relationship is also thought to be bidirectional as depression can both exacerbate and stem from breathlessness through multiple mechanisms.^42^ Further, breathlessness was associated with increased ADL and IADL impairment as in previous work.^43^ Breathlessness was also positively associated with mobility issues,^44^ though this finding may be expected as the mMRC breathlessness measure captures functional limitations, specifically related to walking. This conceptual overlap with the mobility, ADL and IADL measures in the study, means that we cannot fully separate breathlessness from its functional consequences in this cross-sectional analysis. It is plausible that impaired mobility may act as a mediator on the pathway from breathlessness to loneliness, as breathlessness may restrict movement which in turn may affect social contact. Alternatively, mobility may operate as an independent correlate that co-occurs with breathlessness. Longitudinal mediation analyses using temporally separated measures of breathlessness, mobility and loneliness are needed to disentangle these associations.

The overall prevalence of loneliness observed in this ELSA sample (19.3%) is higher than estimates from a cross-sectional analysis of the Health and Retirement Study (HRS; 12%) that focused on loneliness and self-reported COPD.^20^ In our sample, those with grade 2–3 breathlessness were significantly more likely to be lonely than those without breathlessness (no breathlessness, 16.3%; grade 2, 28.4%; grade 3, 34.9%). Similarly, in HRS, loneliness was elevated in those with COPD and further increased in more severe cases (no COPD, 11%; COPD, 18%; COPD on oxygen, 22%). The current analysis extends this evidence by adopting a symptom-based approach in a representative general population sample, a setting where breathlessness remains understudied,^10^ allowing us to capture the burden of breathlessness across diagnoses rather than restricted to a single condition. Those with greater breathlessness were also more likely to live alone, be unmarried and report greater social isolation. This pattern of results is in keeping with the HRS analysis,^20^ reflecting broader deficits in social connectedness in those with breathlessness.

In our sub-analysis of over 1000 participants with grade 2–3 breathlessness, several characteristics were associated with loneliness, many of which reflect well-established predictors of loneliness in other middle-aged and older adult populations. Being unmarried and living alone were among the strongest correlates. This is consistent with prior ELSA research demonstrating the centrality of close social relationships to loneliness^25 28^ and the aforementioned HRS study^20^ where being single was a correlate of loneliness in those with COPD. In contrast, social isolation was not correlated with loneliness in those with breathlessness. This also supports previous ELSA evidence on other physical health conditions,^4 45 46^ suggesting that the quality versus quantity of social connections is differentially associated with health outcomes. Indeed, qualitative work illustrates how people with breathlessness can experience a sense of disconnection or otherness, even in the presence of other people.^47^ Greater depressive symptoms were also strongly associated with loneliness in this group, in line with the HRS results showing depressive symptoms to be a correlate of loneliness in those with COPD.^20^ These findings reflect the well-documented bidirectional relationship between loneliness and depression.^6 48^ This highlights the compounding burden of multimorbidity faced by people managing both breathlessness and mental health issues and potentially highlights that support to address loneliness in this population may require both social and psychological elements.

Female sex was associated with greater loneliness in those with breathlessness, which is consistent with the wider loneliness literature in general population samples.^49–52^ Lower wealth and educational attainment were additionally associated with loneliness, consistent with evidence that socio-economic disadvantage increases the risk of social exclusion in older adults.^3 8 10^ Taken together, these findings highlight the relevance of breathlessness to the NHS England Core20PLUS5 framework’s focus on reducing health inequalities,^53^ which highlights chronic respiratory disease as a priority and recognises that premature mortality from respiratory disease is socio-economically patterned.^54^ Our findings add to this evidence base, suggesting a clustering of multiple disadvantages in this population.

Limiting longstanding illness, mobility impairment and ADL difficulties were also independently associated with loneliness in those with breathlessness. This is similar to previous work, whereby loneliness was correlated with ADL difficulties in those with COPD.^20^ Taken together, these findings and those from qualitative studies^17^ suggest that the social consequences of breathlessness potentially operate partly through its effects on physical function and independence, which is relevant for targeted rehabilitation approaches. Smoking was associated with greater loneliness in this group, aligning with previous work in the wider ELSA sample, demonstrating cross-sectional and longitudinal associations between loneliness and smoking.^55^ It is plausible in those with breathlessness that the correlations between smoking and loneliness could potentially reflect both the social stigma associated with smoking-related breathlessness^14^ and the broader health and social disadvantages that accompany long-term smoking.

Although our cross-sectional design precludes causal conclusions or recommendations about specific clinical actions, the findings highlight some possible avenues for further consideration. Adults with clinically significant breathlessness appear to represent a group in whom loneliness is more prevalent, which may support including loneliness within broader holistic assessment in respiratory, primary care and palliative care settings. Conversely, services addressing loneliness in middle-aged and older adults may benefit from being attentive to physical symptom burden, including breathlessness. Whether symptom-focused approaches (eg, holistic breathlessness services^56^) or loneliness-focused approaches (eg, psychological therapies^57^) influence loneliness in this group warrants exploration; intervention studies that compare symptom-targeted, loneliness-targeted and combined approaches would help to address this question.

This study has several strengths, including the use of a large, nationally representative sample, validated measures of loneliness^23^ and breathlessness^26^ and a comprehensive range of demographic and clinical covariates. Limitations include the cross-sectional design, which precludes causal inference, and the use of data from 2010 to 2011, which may not fully reflect the current burden of breathlessness and loneliness in England. Although recent reviews suggest that the prevalence of breathlessness is similar to figures reported here,^10^ the social and policy context of loneliness has changed since this ELSA data were collected, with the launch of the UK Loneliness Strategy in 2018 and the COVID-19 pandemic, which raised public awareness and altered patterns of social contact. Therefore, the associations reported here should be interpreted as characterising a baseline pre-pandemic period, and replication in more recent data is required. Additionally, while broadly representative of the middle-aged and older adult population, the under-representation of ethnic minority participants in ELSA limits the generalisability of our findings to more diverse populations. The small numbers in each minority ethnic group also preclude meaningful stratified analyses, such as examining whether the breathlessness–loneliness association differs by ethnicity. This is an important area for future work in more ethnically diverse cohorts. Further, as in other analyses of cohort data,^58^ participants who were excluded from the analysis were older and less healthy, which may mean that our results underestimate the true prevalence of loneliness in those with breathlessness due to selection bias arising from missing data. The excluded group (n=2079) differed from the included sample (n=8195) on multiple characteristics including age, ethnicity, wealth, marital status, loneliness, depressive symptoms, limiting longstanding illness, breathlessness and mobility impairment. As the excluded participants were generally more disadvantaged on characteristics associated with both breathlessness and loneliness (ie, older, less wealthy and less healthy), the associations reported here may be conservative estimates of those in the wider population. Logistic regression analyses were restricted to participants with grade 2–3 breathlessness, as mMRC grade ≥2 is the most widely used criterion for clinically significant breathlessness.^10^ While this focused the analysis on adults whose breathlessness symptoms meaningfully restrict everyday activity, it reduced the analytic sample (n=1149) size, which means that our findings on the correlates of loneliness may not generalise to those with milder grade 1 breathlessness. The mMRC measure only captures breathlessness-related functional limitation alone. Further, the measure in ELSA was limited to grades 0–3, meaning those with the most severe breathlessness (grade 4) were not identified separately, potentially leading to further underestimation of how many people with breathlessness are affected by loneliness. Future research should use multidimensional breathlessness measures that capture its sensory, affective and functional components, as well as include participants with more severe breathlessness. The present study was designed as a descriptive, prevalence-focused study. Therefore, we did not formally examine whether the number of co-existing chronic conditions was associated with greater loneliness in those with breathlessness, nor did we model characteristics such as chronic illness, ADL impairment or activity level as mediators of this relationship. These mechanistic questions represent important avenues for future longitudinal mediation studies.

In conclusion, adults living with breathlessness report higher levels of loneliness than those without breathlessness. In this group, loneliness was most strongly associated with being unmarried or living alone, lower wealth, depressive symptoms, limiting longstanding illness and mobility and ADL impairment. As these associations are cross-sectional, they should be interpreted as markers of higher loneliness risk rather than as targets for intervention; longitudinal research is needed to clarify directionality before clinical implications can be drawn. Future longitudinal research should explore the pathways linking breathlessness and loneliness, including potential mediators such as comorbidity burden, depressive symptoms, mobility and ADL impairment, to inform potential future interventions that may prevent or reduce loneliness.

## Supplementary material

10.1136/bmjresp-2026-004357 online supplemental file 1

## Data Availability

Data are available in a public, open access repository.
