# Supplementary material for: Prevalence of loneliness and its associated factors in middle-aged and older adults with breathlessness: a nationally representative cohort study
Source: BMJ Open Respir Res. 2026 Jul 29;13(1):e004357. doi: 10.1136/bmjresp-2026-004357 (PMC13423144; doi:10.1136/bmjresp-2026-004357)
Supplement: online supplemental file 1 [file bmjresp-13-1-s001.pdf]

Prevalence of loneliness and its associated factors in middle-aged and older adults with breathlessness: a nationally representative cohort study (Hackett et al.) – Supplementary File

Table S1: Characteristics of sample by breathlessness grade at wave 5 (2010/11) of the English Longitudinal Study of Ageing (**weighted**)

	**Total sample (N=7307)**	**No breathlessness (n=5165)**	**Grade 1 breathlessness (n=1063)**	**Grade 2 breathlessness (n=332)**	**Grade 3 breathlessness (n=747)**	***p***
Age (years)	66.51 (8.71)	65.36 (8.34)	68.73 (8.70)	68.44 (8.77)	70.63 (9.29)	**< 0.001**
Sex (% female)	840 (52.6%)	2529 (49.0%)	675 (63.5%)	214 (64.5%)	422 (56.5%)	**< 0.001**
Ethnicity (% yes)[a]	-	-	-	-	-	0.116
- White	7079 (96.9%)	5020 (97.2%)	1029 (96.7%)	317 (95.8%)	713 (95.4%)	
- Mixed ethnicity	14 (0.2%)	7 (0.1%)	2 (0.2%)	2 (0.6%)	3 (0.4%)	
- Black	66 (0.9%)	41 (0.8%)	12 (1.1%)	2 (0.6%)	11 (1.5%)	
- Asian	105 (1.4%)	64 (1.2%)	18 (1.7%)	7 (2.1%)	16 (2.1%)	
- Other	42 (0.6%)	32 (0.6%)	3 (0.3%)	3 (0.9%)	4 (0.5%)	
Wealth quintile (£)[b]	-	-	-	-	-	**< 0.001**
- 1	1221 (17.0%)	622 (12.3%)	238 (22.7%)	80 (24.2%)	281 (38.1%)	
- 2	1434 (19.9%)	920 (18.1%)	247 (23.5%)	81 (24.5%)	186 (25.2%)	
- 3	1444 (20.1%)	1020 (20.1%)	220 (21.0%)	81 (24.5%)	123 (16.7%)	
- 4	1519 (21.1%)	1183 (23.3%)	185 (17.6%)	58 (17.6%)	93 (12.6%)	
- 5	1570 (21.8%)	1326 (26.1%)	159 (15.2%)	30 (9.1%)	55 (7.5%)	
Education (% yes)[c]	-	-	-	-	-	**< 0.001**
- University degree	1341 (18.3%)	1143 (22.1%)	110 (10.3%)	34 (10.2%)	54 (7.2%)	
- A-levels/O-levels	4073 (55.7%)	2954 (57.2%)	592 (55.6%)	183 (55.0%)	344 (46.0%)	
- No qualifications	1894 (25.9%)	1066 (20.6%)	362 (34.0%)	116 (34.8%)	350 (46.8%)	
Marital status (% yes)[d]	-	-	-	-	-	**< 0.001**
- Married	5046 (69.0%)	3761 (72.8%)	668 (62.8%)	219 (65.8%)	398 (53.2%)	
- Never married	431 (5.9%)	316 (6.1%)	59 (5.6%)	13 (3.9%)	43 (5.7%)	
- Divorced	868 (11.9%)	563 (10.9%)	140 (13.2%)	42 (12.6%)	123 (16.4%)	
- Widowed	964 (13.2%)	525 (10.2%)	196 (18.4%)	59 (17.7%)	184 (24.6%)	
UCLA loneliness (SD)	4.14 (1.51)	3.99 (1.40)	4.25 (1.52)	4.56 (1.69)	4.87 (1.87)	**< 0.001**
UCLA loneliness ≥ 6 (%)	1474 (20.2%)	880 (17.0%)	225 (21.2%)	97 (29.2%)	272 (36.4%)	**< 0.001**
Loneliness one item (% yes)[e]	508 (7.0%)	250 (4.8%)	78 (7.4%)	38 (11.4%)	142 (19.1%)	**< 0.001**
Social isolation (no marital status)[f]	-	-	-	-	-	**0.006**
- 0	812 (14.1%)	575 (14.4%)	123 (14.4%)	27 (10.2%)	86 (14.0%)	
- 1	3070 (53.5%)	2189 (54.7%)	451 (52.9%)	138 (52.1%)	292 (47.5%)	
- 2	1456 (25.4%)	971 (24.3%)	222 (26.0%)	78 (29.4%)	185 (30.1%)	
- 3	400 (7.0%)	268 (6.7%)	57 (6.7%)	22 (8.3%)	52 (8.5%)	
Living alone (% yes)	1616 (22.1%)	949 (18.4%)	303 (28.5%)	85 (25.6%)	279 (37.3%)	**< 0.001**
CES-D depression (SD)[g]	1.38 (1.86)	1.07 (1.61)	1.60 (1.93)	2.37 (2.22)	2.82 (2.38)	**< 0.001**
CES-D (without loneliness) (SD)[g]	1.26 (1.70)	0.98 (1.48)	1.46 (1.74)	2.19 (2.02)	2.55 (2.13)	**< 0.001**
Limiting illness (% yes)	2225 (30.5%)	1023 (19.8%)	409 (38.5%)	219 (66.0%)	575 (77.0%)	**< 0.001**
Coronary heart disease (%yes)	932 (12.8%)	402 (7.8%)	194 (18.3%)	75 (22.6%)	261 (34.9%)	**< 0.001**
Stroke (% yes)	253 (3.5%)	112 (2.2%)	41 (3.9%)	22 (6.6%)	78 (10.4%)	**< 0.001**
Diabetes (% yes)	761 (10.4%)	410 (7.9%)	132 (12.4%)	60 (18.1%)	159 (21.3%)	**< 0.001**
Lung disease (% yes)	363 (5%)	71(1.4%)	69 (6.5%)	55 (16.6%)	168 (22.5%)	**< 0.001**
Asthma (% yes)	843 (11.5%)	361 (7.0%)	198 (18.6%)	64 (19.3%)	220 (29.5%)	**< 0.001**
Arthritis (% yes)	2693 (36.9%)	1547 (30.0%)	488 (45.9%)	205 (61.7%)	453 (60.6%)	**< 0.001**
Cancer (% yes)[h]	430 (5.9%)	257 (5.0%)	71 (6.7%)	26 (7.8%)	76 (10.2%)	**< 0.001**
Smoking status[i]	–	–	–	–	–	**< 0.001**
- Never	6109 (83.6%)	4409 (85.4%)	859 (80.9%)	271 (81.6%)	570 (76.4%)	
- Former	239 (3.3%)	179 (3.5%)	23 (2.2%)	11 (3.3%)	26 (3.5%)	
- Current	957 (13.1%)	577 (11.2%)	180 (16.9%)	50 (15.1%)	150 (20.1%)	
ADL (SD)[j]	0.25 (0.74)	0.11 (0.48)	0.28 (0.69)	0.64 (1.13)	1.02 (1.33)	**< 0.001**
IADL (SD)[j]	0.29 (0.80)	0.14 (0.55)	0.30 (0.79)	0.67 (1.04)	1.14 (1.39)	**< 0.001**
Mobility issues (SD)[k]	1.66 (2.29)	0.94 (1.63)	2.16 (2.07)	3.99 (2.50)	5.00 (2.67)	**< 0.001**

Data presented as means (SD) and n (%). Percent is valid percent.

ADL= Activities of daily living; CES-D= Center for Epidemiologic Studies Depression Scale; IADL= Instrumental activities of daily living

[a] N = 7306 Ethnicity; [b] N = 7188 Wealth; [c] N = 7308 Education; [d] N = 7309 Marital status; [e] N = 7302 Single-item loneliness
[f] N = 5738 Social isolation; [g] N = 7909 CES-D; [h] N = 7306 Cancer; [i] N = 7305 Smoking status; [j] N = 7936 ADL/IADL; [k] N = 7935 Mobility issues
